# Determination of puberty in gilts: contrast of diagnostic methods

**DOI:** 10.1186/s40813-022-00271-0

**Published:** 2022-06-16

**Authors:** Antonio Vela, Andrés Suárez-Usbeck, Laura Lafoz, Olga Mitjana, María Teresa Tejedor, Sofía Martín, Marina López, María Victoria Falceto

**Affiliations:** 1 THINKINPIG S.L., Avenida Gómez Laguna, 41 10ºA, 50009 Zaragoza, Spain; 2 Department of Animal Pathology, Reproductive and Obstetric Area, Faculty of Veterinary Medicine, Agroalimentary Institute of Aragon-IA2, Universidad de Zaragoza-CITA, Zaragoza, Spain; 3 Department of Anatomy, Embryology and Animal Genetics, CIBERCV, Genetics Area, Faculty of Veterinary Medicine, Universidad de Zaragoza, Zaragoza, Spain; 4 KUBUS Calle Varsovia, 20, 28232 Las Rozas de Madrid, Madrid, Spain

**Keywords:** Puberty diagnosis, Gilts, Multiple logistic regression analysis, Ultrasonography, Sensibility, Specificity, Positive predictive value, Negative predictive value

## Abstract

**Background:**

Early onset of a gilt´s puberty is needed for adequate economic performance in farms, because it indicates her reproductive performance and longevity. Therefore, an effective diagnosis is needed. Our purpose was to compare different procedures (external characteristics, blood progesterone analysis and ultrasonography diagnosis) to detect puberty in 70 gilts (Topigs TN70; 240 days old) on farm conditions. Postmortem examination was the standard reference. Multiple logistic regression analysis was used to identify which combination of independent variables (predictors) best predicts the status of gilts.

**Results:**

Puberty (46/70 gilts; 65.71%) was characterized by the presence of follicles larger than 6 mm, *corpus albicans, corpus rubrum,* and *corpus luteum* (postmortem examination). Vaginal length, body condition, backfat, carcass weight and progesterone blood concentration were significantly higher in pubertal than prepubertal gilts (*P* < 0.05). Two types of ultrasonography equipment (DELTA and W3) were compared and performed by the same senior technician (V1). The results obtained by two technicians with different levels of experience (V1 and V2, a junior technician) using W3 were also compared. Ultrasonography provided better results than other diagnostic techniques, although the effectiveness of the ultrasonography changed with technological improvements and with increased expertise of technicians. The most accurate results were found by V1/DELTA (Nagelkerke´s R^2^ = 0.846; Sensitivity = 0.956; Specificity = 0.958; Positive predictive value = 0.978; Negative predictive value = 0.920; Area under ROC curve = 0.957). Results using the W3 equipment could be improved when used in conjunction with vaginal length (V1; Nagelkerke´s R^2^ = 0.834; Sensitivity = 0.933; Specificity = 0.958; Positive predictive value = 0.977; Negative predictive value = 0.885; Area under ROC curve = 0.972) or progesterone concentration (V2; Nagelkerke´s R^2^ = 0.780; Sensitivity = 0.955; Specificity = 0.826; Positive predictive value = 0.915; Negative predictive value = 0.905; Area under ROC curve = 0.970).

**Conclusions:**

Ultrasonography provided better results than other diagnostic techniques. The effectiveness of the ultrasonography changes with technological improvements and with increased expertise of technicians. Results using the W3 equipment could be improved when used along with vaginal length (V1) or progesterone concentration (V2). Accuracy parameters are a guide to choose puberty diagnosis, but the farms must also evaluate effect on gilts, ease and cost of administration.

## Background

Gilt productivity is important in pork production enterprises because gilts account for 20–25% of the farrowing group. In most farms, 30%-50% of the sow herd is annually replaced. Therefore, an efficient reproduction control greatly impacts on the overall production [1, 2]. Early onset of gilt puberty is needed for adequate economic performance in commercial pig farms [3]. In fact, almost 10% of gilts are slaughtered before their first artificial insemination (AI), mainly due to reproductive problems [4]. Gilts reach puberty between 150 and 220 days-of-age [5]; therefore, accurate detection of first estrus is key to optimize the correct time of AI in their second or third estrus [6]. The onset of puberty is a reliable indicator of gilt reproductive performance and longevity [7]. The first estrus involves the efficiency of genetic potential and the physiological mechanisms that affect the sexual maturation and reproductive management in gilts [8].

Reproductive failure by delayed puberty after 7–8 months was reported as the main reason for discarding gilts [9, 10]. An increase in the age at first AI (220–300 days) was associated with 2.1% increased risk of culling due to fertility failures [8, 10]. Up to 30–40% of gilts older than 8 months did not show any sign of estrus and consequently were culled [10]. Silent estrus (ovulation without sign of estrus) occurs only in 4–5% of gilts [11]; this silent estrus may be due to an underdeveloped hypothalamic-pituitary axis without a positive feedback response by low blood estrogen concentrations [12]. However, the evidence of undetectable estrus in gilts highlights the importance of an effective reproductive management program [8]. Postmortem examination of reproductive organs showed that 60% of gilts supposed to be in anestrus had cyclical ovarian activity [8, 11, 13, 14]. Therefore, an anestrus diagnosis in gilts might be due to an inadequate estrus detection, rather than an actual absence of physiological estrus [5, 11, 14].

Currently, puberty is determined on farms by means of boar exposure and detection of signs of estrus (swollen and red vulva, interest ion boars and standing reflex in response to back pressure) [15]. Other methods proposed for detecting sexual maturity in gilts include blood progesterone analysis [16, 17], laparoscopy [18] and postmortem examination [19]. These techniques are expensive and, increase farm work and/or cause damage to animals; therefore, they are not routinely used in farms.

Certain external characteristics would be useful in detecting puberty. The study of the reproductive tract of the gilt from birth to puberty showed important changes in the weight and length of the oviducts and the uterus when puberty was reached [20]. Recently, the length of vagina-cervix has been related to length and capacity of uterine horns [21, 22]. A certain level of body condition would be needed for puberty onset in gilts [23]; also, fat mass was associated with puberty in female mammals [24]. Growth patterns, easily measurable in farm, would be predictive of puberty onset in gilts [25]; therefore, the length of vagina-cervix, body condition and backfat would be valuable in detecting puberty. In sows, ultrasonography has proved to be useful in detecting pregnancy, estimating time of ovulation and determining ovarian pathology [26]. Also, ultrasonography allows visualization of gilt uterus and ovaries and is considered highly sensitive for puberty diagnosis [27–30].

The purpose of this study was to compare different procedures (external characteristics, blood progesterone analysis and ultrasonography diagnosis) in terms of their ability to detect puberty in gilts on farm conditions. Moreover, two types of ultrasonography equipment were compared when used by the same technician. Also, the results from two technicians with different levels of experience were compared when using the same equipment. Postmortem examination was the standard reference.

## Materials and methods

### Animals

This study was performed in accordance with the European Directive for pig protection [31] and the Spanish legislation for animal protection in experimentation and other scientific purposes, including teaching [32]. Expert veterinarians were in charge of caring and handling the animals. The Ethical Committee for Animal Experiments, University of Zaragoza, Spain approved this study (reference number: PI01/22).

This study was conducted according to the Spanish standard commercial swine production on a breed farm located near Tarragona (La Horta de Sant Joan, South-eastern Spain). Out of a total of 400 gilts housed in 40 pens (10 gilts/pen, pen size: 2 × 5 m), 70 gilts (Topigs TN70, Topigs Norsvin, Madrid, Spain) were randomly chosen to use in the study; these gilts were 240 days old. Gilts were not previously exposed to boars nor was estrus previously checked by behavioral characteristics. No estrus-stimulating treatment was performed. Several reasons explained for these decisions in the study design. We intended to contrast several puberty diagnostic methods as blind tests. Moreover, our previous experience showed that at 240 days of age the gilts could be pubertal or not, and the detection of their pubertal status was the basis for this contrast of methods. Gilts were fed ad libitum with a commercial finishing diet containing 3200 kcal/kg metabolizable energy (ME), 15.9% crude protein (CP), and 1.19% digestible lysine. Also, water was available ad libitum.

### Puberty diagnosis

The results from several diagnostic methods were blindly assessed to assure their independence from verified gilts status according to the reference standard. Each diagnostic method of this blind study was performed by different researchers. The measurement of the external characteristics and the extraction of blood for the quantification of serum progesterone (P4) were carried out simultaneously. Later on the same day, the ultrasonography was carried out. All gilts were slaughtered the day after the scan (16 h afterwards).

### External characteristics

Farm diagnosis of puberty is based on several external characteristics, easily measurable on gilts in farm condition. Vaginal length (cm) was measured using a calibrated catheter (KUBUS, Madrid, Spain). Body condition was evaluated by visual scoring on a scale ranging from 1 to 5: 1 was used for extremely thin sows and 5 for extremely fat ones [33]. Backfat measurements (mm) were performed using the P2 method [9].

Individual live weight was not measured but it was estimated from carcass weight, by using the following formula**:** Live weight = hot carcass weight/typical dressing percentage, where typical dressing percentage was set at 70% [34].

### Progesterone concentration

Blood sample collection (10 ml) was individually performed by jugular venipuncture using sterile tubes without additives (Vacutainer Brand, Devon, UK).

Blood serum progesterone concentrations (P4) were assessed in an external laboratory by a P4 analytical method PNT-HOR-30409, ELFA (Laboratorios CONVET S.L., Lleida, Spain).

### Ultrasonography

The gilts were submitted to transcutaneous ultrasonography in their pens; both ultrasonography equipment are portable. In a first phase of the study, every gilt was studied by a senior, expert technician (V1) using the high-resolution ultrasound Mylab Delta® (Esaote, Barcelona, Spain), adjusted to 8.6 MHz microconvex transducer (henceforth Delta). In a second phase, all gilts were successively and independently scanned using the same equipment by two technicians with different levels of expertise: V1 (senior) and V2 (junior). The ultrasound equipment used by both technicians in this second phase was a commercial ultrasound W3® (KUBUS, Madrid, Spain) adjusted to a 3.5 MHz wireless sectorial transducer (henceforth W3). In total, three scans were conducted on every gilt.

Ultrasound puberty diagnosis was performed according to a modified procedure described by Kauffold et al. [30], based on the evaluation of uterus size and position and the visualization and analysis of the ovary. The transducer was placed horizontally on the right or left ventro-lateral abdominal wall just dorsal to the last pair of teats.

Sexual maturity was expressed as ‘‘prepubertal” (PRE) and ‘‘pubertal’’ (PUB) according to the following criteria, that must be fulfilled simultaneously:

1. *Uterus*: Gilts were classified as PRE when during the scan of the bladder, the total volume space occupied by the uterus in its widest section is ≤ 1/3 total of the ultrasound section on the screen. Identification of the bladder is necessary for a proper assessment. Instead, gilts were classified as PUB when the total volume space occupied by the uterus in its widest section (the bladder may or may not appear in the image) is ≥ 2/3 total ultrasound section on the screen.
2. *Uterine horns*: The uterine horns were scanned in cross-sections. When the measured digital strip was ≥ 1cm^2^, gilts were classified as PUB; otherwise, they were considered as PRE.
3. *Ovary*: The PRE gilts shows a major ovarian diameter ≤ 2.5–3 cm with obvious connective tissue, seen as hyperechoic lines holding < 4 mm follicles. In follicular phase (PUB gilts), follicular size varies from 4 to 8.5 mm, based on timing of ultrasonography relative to ovulation. In proestrus, follicular size varies 5–5.5 mm and in estrus, follicular size exceed 5.5 mm. In metaestrus, corpus rubrum occurs and in diestrus, corpus luteum (5–10 mm) appears.

The time required for every ultrasound procedure/technician was also recorded.

### Postmortem examination

The postmortem examination provides the reference standard to which the accuracy of the other diagnostic methods was stablished. Gilt status (PRE/PUB) was assessed on the basis of postmortem study of the genital tract with special emphasis on the ovarian structures [11]. PUB status was characterized by presence of follicles larger than 6 mm, *corpus albicans* (PUB in proestrus-estrus), *corpus rubrum* (PUB in metaestrus) and *corpus luteum* (PUB in diestrus). The absence of these structures (*corpus albicans, corpus rubrum* and *corpus luteum*) pointed to PRE status.

Also, the genital tract was dissected and the morphometry of each part was separately analyzed: dimensions (cm) and weight (g) of ovaries, oviducts, uterine horns and body were recorded. The absence of cervix and vagina in postmortem collected at the slaughterhouse prevents us from having these data**.** Carcass weight was also recorded.

### Statistical analysis

Statistical analyses were performed by using IBM SPSS version 26 software (SPSS, Chicago, IL, USA). Means and standard deviation (SD) summarize the quantitative variables (morphometric data, weight, backfat, body condition, P4, and times for ultrasound procedures) and counts of *corpus rubrum*, luteum and albicans.

Follicles sizes were grouped in semi-open intervals starting from the first category (≤ 1 mm). Interval semi-open on the left (a, b] is the set of all real numbers greater than “a” and less than or equal to “b”. In this way, eight categories for follicles size were created. For every individual, percentage of each category was calculated on total follicles number. Means and SD were also estimated for each category.

One–way ANOVA (analysis of variance) was applied to comparisons between PRE and PUB groups for carcass weight and backfat. Comparisons between groups for morphometric variants were carried out by ANCOVA (analysis of covariance) where carcass weight was included as covariate. A non-parametric test (Mann–Whitney U test) compared distribution of follicles size intervals, body condition and P4 between groups. Friedman test (non-parametric) was used to compare needed time among ultrasound procedures.

Cohen´s κ was run to determine if there was agreement in the ultrasound test results from the same individuals in two situations: (1) two types of equipment (Delta and W3) used by one technician (V1) and (2) the same equipment (W3) used by two technicians (V1 and V2). As usual, the greater the value of κ, the greater the strength of the agreement (< 0.20: poor; 0.21–0.40: weak; 0.41–0.60: moderate; 0.61–0.80: good; 0.81–1.00; very good) [35].

Multiple logistic regression analysis was used to identify which combination of independent variables (predictors) best predicts the status of gilts (dependent variable: PRE or PUB). A stepwise procedure (Method: forward) was applied; independent variables moved in or out of the model at any step of the process, on the basis of the Wald test, which determined statistical significance for each of the independent variables: the significance levels to enter and to be removed were *P* ≤ 0.05 and *P* ≥ 0.10, respectively [36]. When ultrasound procedure results were considered as independent variable, the reference level was PRE (coded as 0), to which the other one (PUB, coded as 1) will be compared. Model fit was assessed by chi squared test (omnibus test of model coefficients) that provides the overall statistical significance of the model. Nagelkerke R^2^ estimates how much variation in the dependent variable can be explained by the model. A cut–off point of 0.5 was used and the gilt status will be classified as PUB only if its predicted probability was ≥ 0.5. The ability of models to discriminate between PRE or PUB individuals was assessed by estimating sensitivity (true positive rate), specificity (true negative rate), positive and negative predictive values (proportions of positive and negative results in diagnostic tests that are true positive and true negative results, respectively) [37]. The area under the Receiver Operating Characteristics (ROC) curve estimates an overall measure of discrimination [38].

*P* values < 0.05 were considered as statistically significant.

## Results

**Fig. 1:**
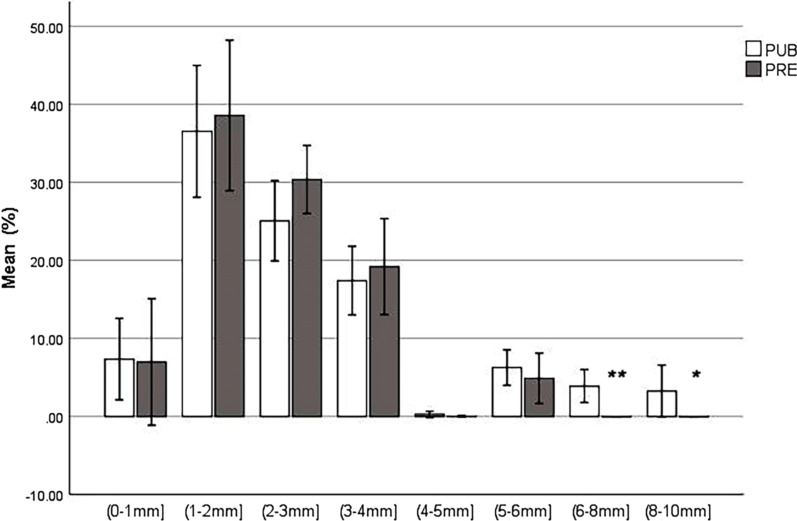
Distribution of follicles size intervals (%) from postmortem examination. Error bars: 95% CI;*: *P* < 0.05; **P < 0.001

Puberty was assessed in 46/70 (65.71%) studied gilts by means of postmortem examination (reference standard). Distribution of follicle size intervals are shown in Fig. 1.

Significant differences (*P* < 0.05) were found among PRE and PUB groups for percentage of follicles larger than 6 mm. Number of *corpus rubrum*, *corpus luteum* and *corpus albicans* in PUB gilts are showed in Table 1.

**Table 1:** Number of corpus rubrum, luteum and albicans in PUB gilts (Mean ± SD)

Ovary side and *corpus*	Mean ± SD
Left ovary corpus *rubrum*	3.15 ± 4.269
Right ovary *corpus rubrum*	3.57 ± 4.778
Left ovary *corpus luteum*	4.61 ± 5.931
Right ovary *corpus luteum*	3.98 ± 4.842
Left ovary *corpus albicans*	9.09 ± 6.390
Right ovary *corpus albicans*	8.20 ± 5.837

The carcass was significantly heavier (*P* < 0.001) in the PUB group (105.12 ± 14.666 kg) than in the PRE group (91.17 ± 15.189 kg). Table 2 shows dimensions and weight of genitalia from postmortem examination. Significant differences were found in every case, with higher dimensions and weight in the PUB group (*P* < 0.001).

**Table 2:** Morphometry of genital tract from postmortem examination (Mean ± SD)

Trait	PRE (n = 24)	PUB (n = 46)	*P V*alue
Right ovary thickness (cm)	1.09 ± 0.230	1.59 ± 0.401	< 0.001
Left ovary thickness (cm)	1.11 ± 0.238	1.57 ± 0.392	< 0.001
Right ovary height (cm)	2.05 ± 0.209	2.64 ± 0.466	< 0.001
Left ovary height (cm)	2.15 ± 0.257	2.75 ± 0.540	< 0.001
Right ovary length (cm)	2.95 ± 0.365	3.82 ± 0.511	< 0.001
Left ovary length (cm)	2.99 ± 0.322	3.94 ± 0.697	< 0.001
Right ovary weight (g)	3.21 ± 0.815	5.94 ± 2.262	< 0.001
Left ovary weight (g)	3.53 ± 0.923	6.65 ± 2.868	< 0.001
Right oviduct length (cm)	20.22 ± 2.280	28.24 ± 3.787	< 0.001
Left oviduct length (cm)	21.23 ± 3.159	30.11 ± 3.707	< 0.001
Right oviduct weight (g)	1.01 ± 0.302	2.07 ± 0.536	< 0.001
Left oviduct weight (g)	1.05 ± 0.308	2.11 ± 0.508	< 0.001
Right uterine horn diameter (cm)	1.72 ± 0.431	2.76 ± 0.512	< 0.001
Left uterine horn diameter (cm)	1.72 ± 0.431	2.80 ± 0.572	< 0.001
Right uterine horn length (cm)	64.73 ± 11.536	128.71 ± 30.230	< 0.001
Left uterine horn length (cm)	68.33 ± 12.269	131.91 ± 33.030	< 0.001
Right uterine horn weight (g)	42.61 ± 22.293	251.64 ± 90.360	< 0.001
Left uterine horn weight (g)	43.38 ± 22.694	245.25 ± 91.348	< 0.001
Right uterine horn thickness (cm)	0.13 ± 0.086	0.42 ± 0.139	< 0.001
Left uterine horn thickness (cm)	0.13 ± 0.086	0.42 ± 0.139	< 0.001
Uterine body length (cm)	2.59 ± 0.757	3.89 ± 0.843	< 0.001
Uterine body weight (g)	3.06 ± 1.410	11.38 ± 4.268	< 0.001

Results for the parameters measured on the farm (vaginal length, body condition, backfat, and estimated live weight) and P4 are shown in Table 3. This table also shows data for progesterone concentrations. Pubertal gilts always showed higher values (*P* < 0.05).

**Table 3:** Farm parameters and progesterone concentration (Mean ± SD)

Traits	PRE (n = 24)	PUB (n = 46)	*P* Value
Vaginal length (cm)	21.75 ± 3.848	27.35 ± 5.435	0.003
Body condition (1–5 pts)	3.13 ± 0.338	3.43 ± 0.501	< 0.001
Backfat (mm)	6.46 ± 1.931	9.50 ± 2.469	< 0.001
Live weight (kg)	130.24 ± 21.693	150.16 ± 20.970	< 0.001
P4 (ngmL^−1^)	0.84 ± 0.414	29.10 ± 27.941	< 0.001

**Fig. 2:**
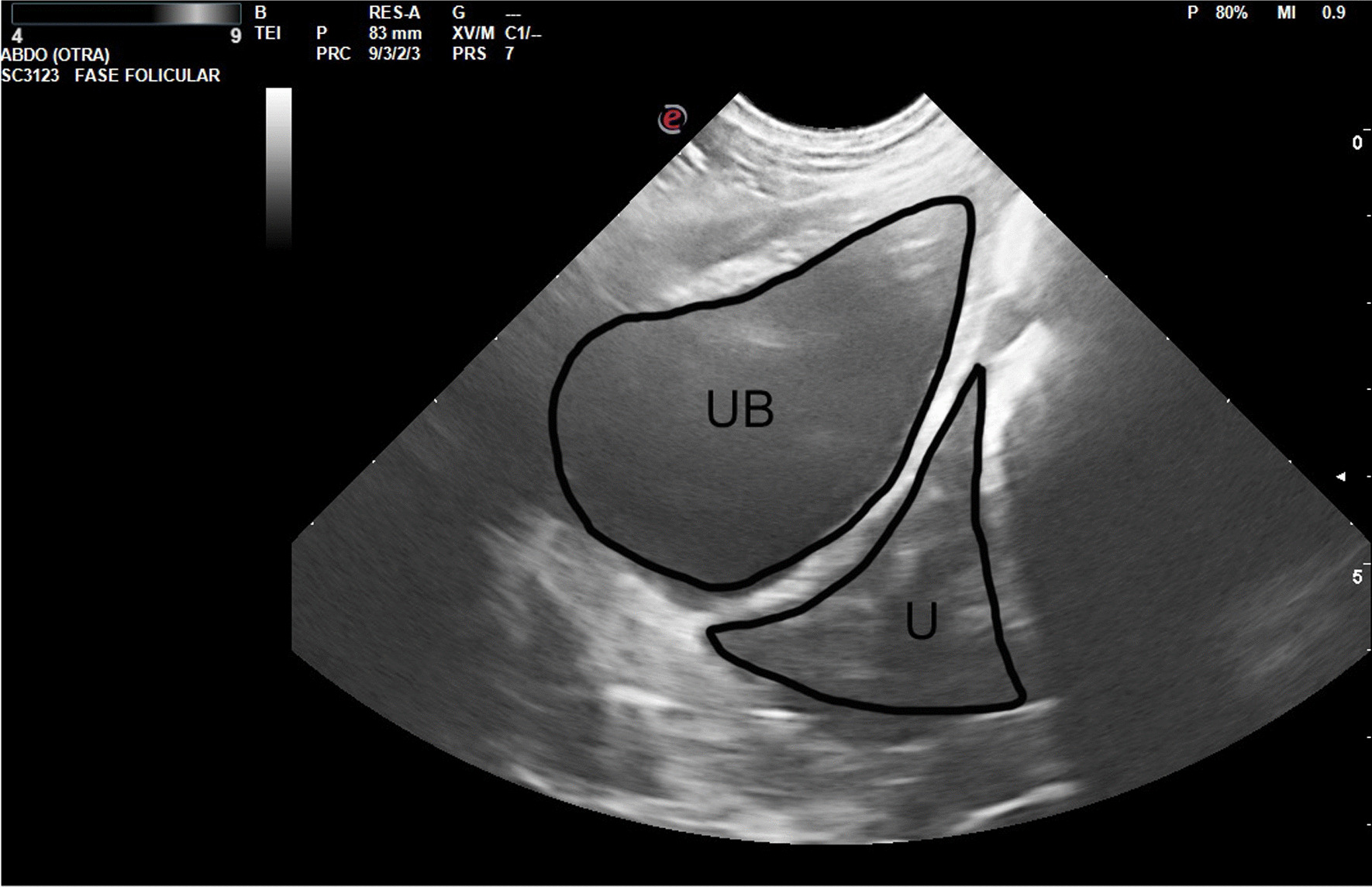
Prepubertal gilt (V1/Delta). The urine bladder (UB) appears as an anechoic structure in the center of the image, just below the small uterus (U), well delimited by the intestinal loops

**Fig. 3:**
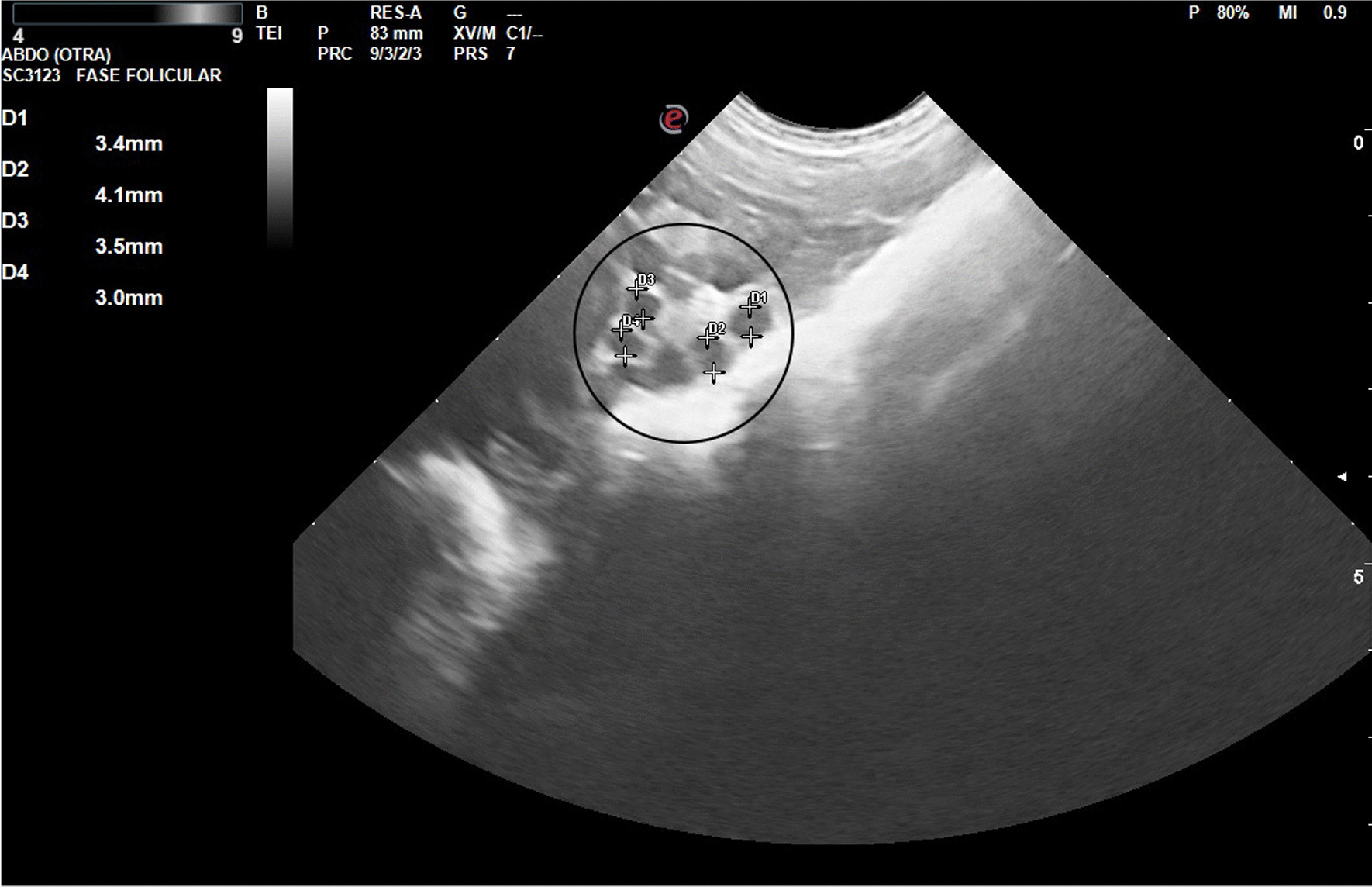
Prepubertal gilt (V1/Delta). Small ovary (2.7 cm) and follicles (2–4 mm; inside the circle)

**Fig. 4:**
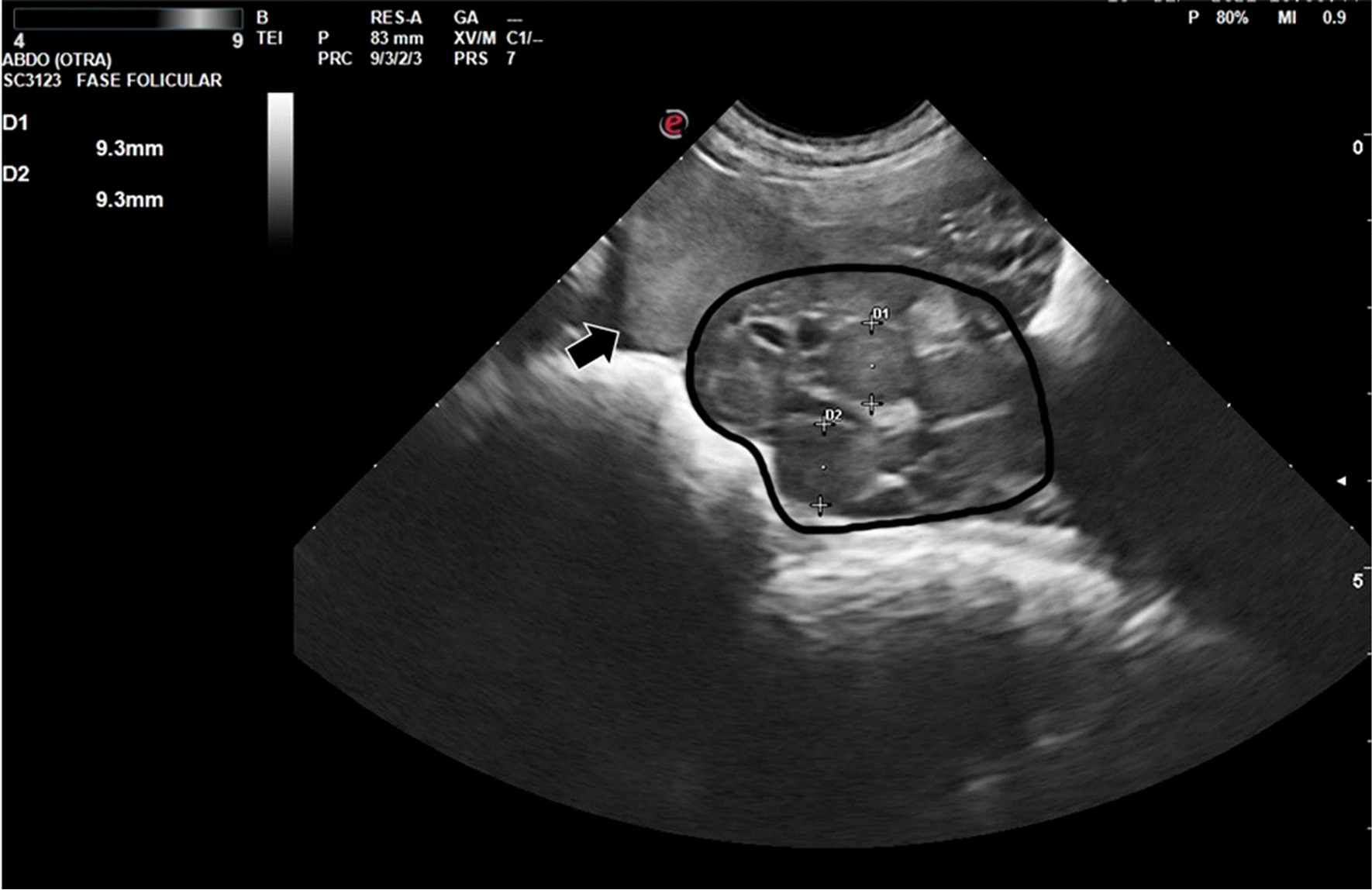
Pubertal gilt (V1/Delta). Inside the circle: ovary with corpora lutea. Five corpora lutea are clearly visible; one more corpus luteus would be occult. Two of them were measured (approximately 9 mm each diestrum middle phase). The intestinal loops can be seen under the ovary. The arrow signals the section of a uterine horn (diameter: 2 cm)

**Fig. 5:**
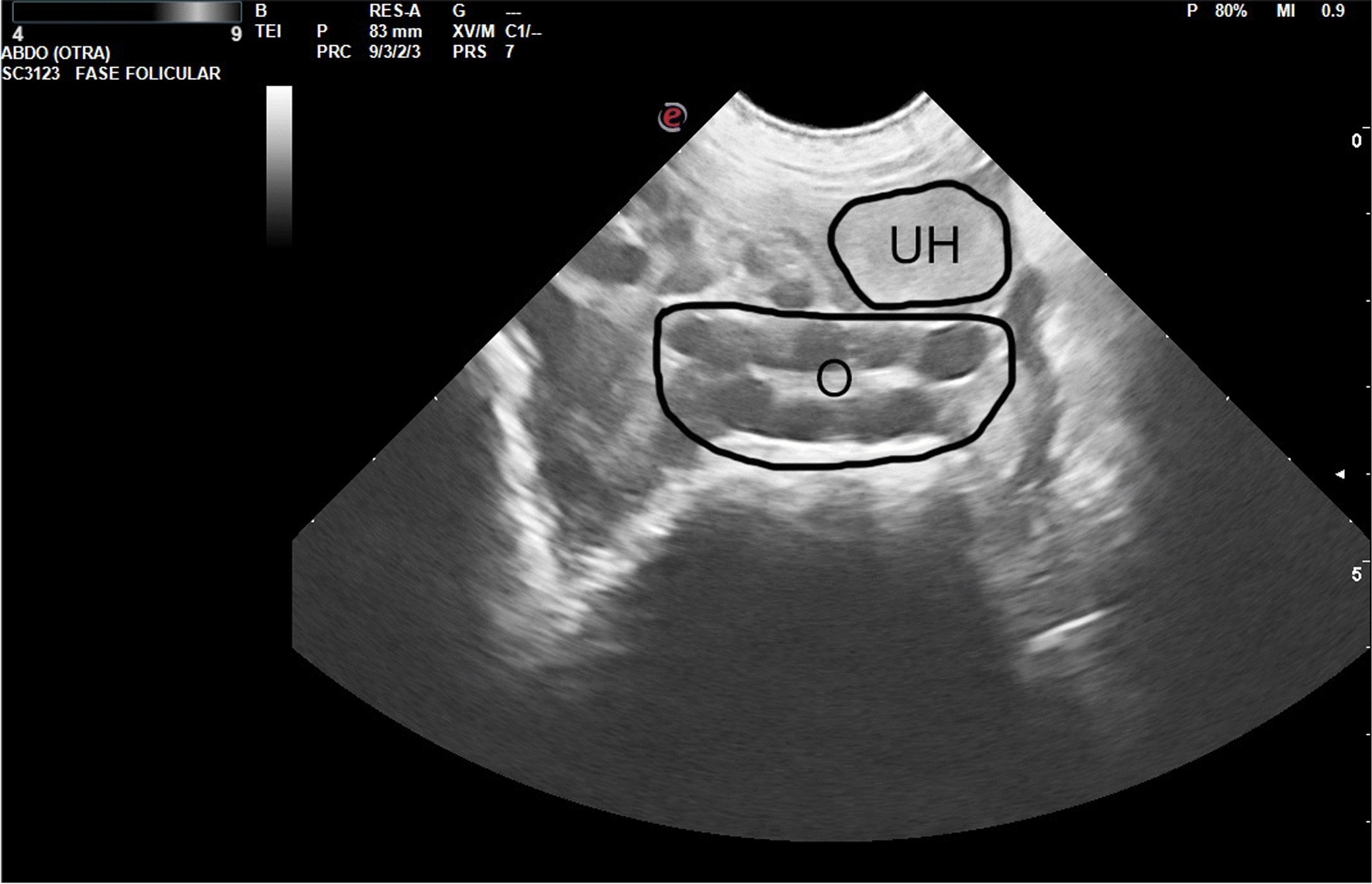
Pubertal gilt (follicular phase, V1/ Delta). The ovary (O) with preovulatory follicles can be seen in the center of the image. Above the ovary, the section of a uterine horn (UH) can be seen (diameter: 2 cm). Under the ovary, the image shows the intestinal loops

Figures 2, 3, 4 and 5 show ultrasonography images from V1/Delta. It is noteworthy that explaining an echography by an only image is very difficult. Usually, one frame is selected, where the structure under study is represented.

In Fig. 5, an ovary is clearly visible in the center of the image. Around it, there is a venous plexus than could be confused with follicles in a still image like this; a moving image would clearly capture the difference between these structures.

**Fig. 6:**
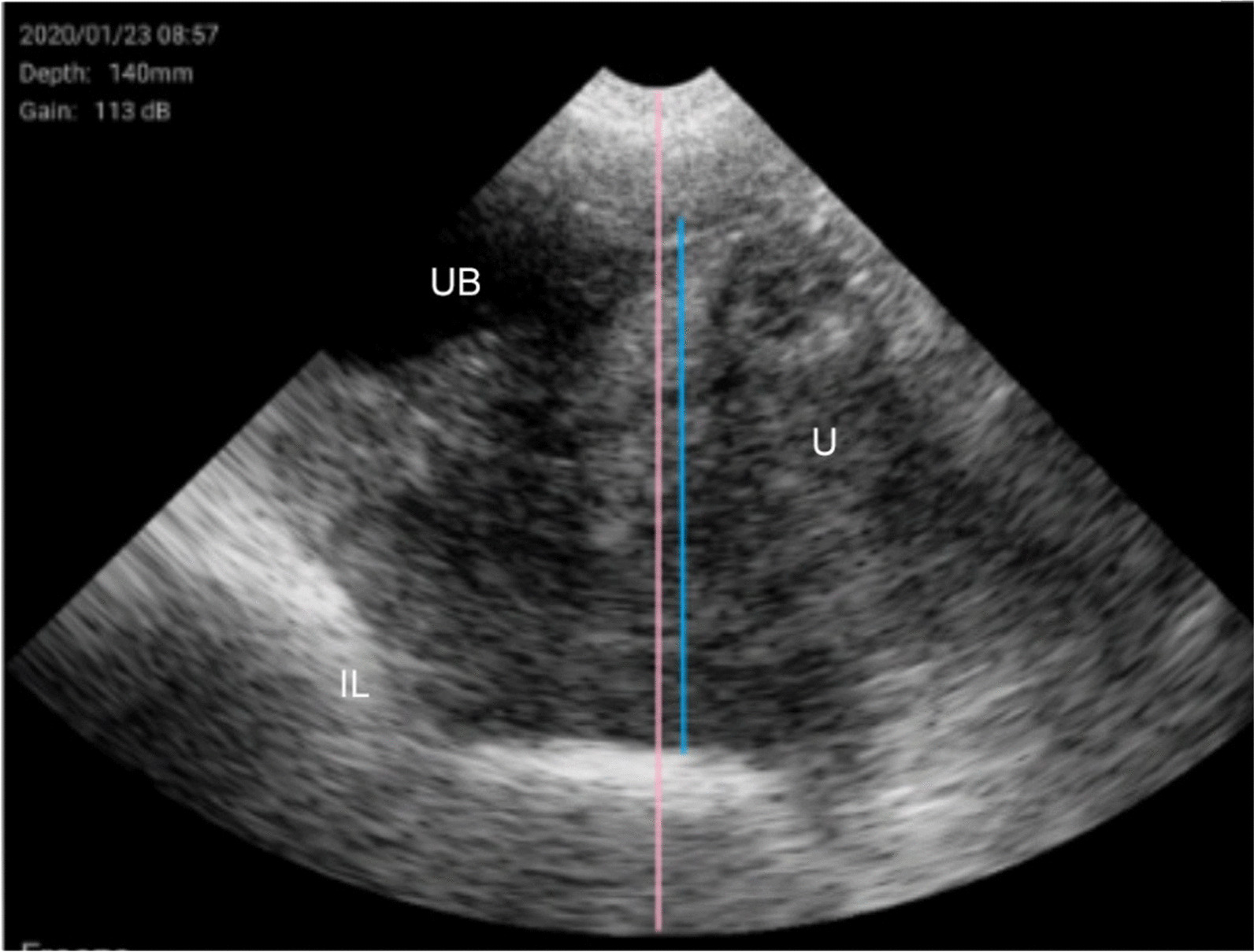
Pubertal gilt (V2/W3). Urine bladder (UB), intestinal loop (IL) and uterus (U) appear as well distinguishable structures. UB shows a completely anechoic structure typical of liquids. IL is characterized by the gas hyperechogenicity. U is a central, homogeneous and echogenic structure situated below and in front of UB; it occupies almost the entire screen. The U height (in blue) is greater than two-thirds of the total height of the image (in pink), which highlights the large volume of the uterus, characteristic of a pubertal gilt

**Fig. 7:**
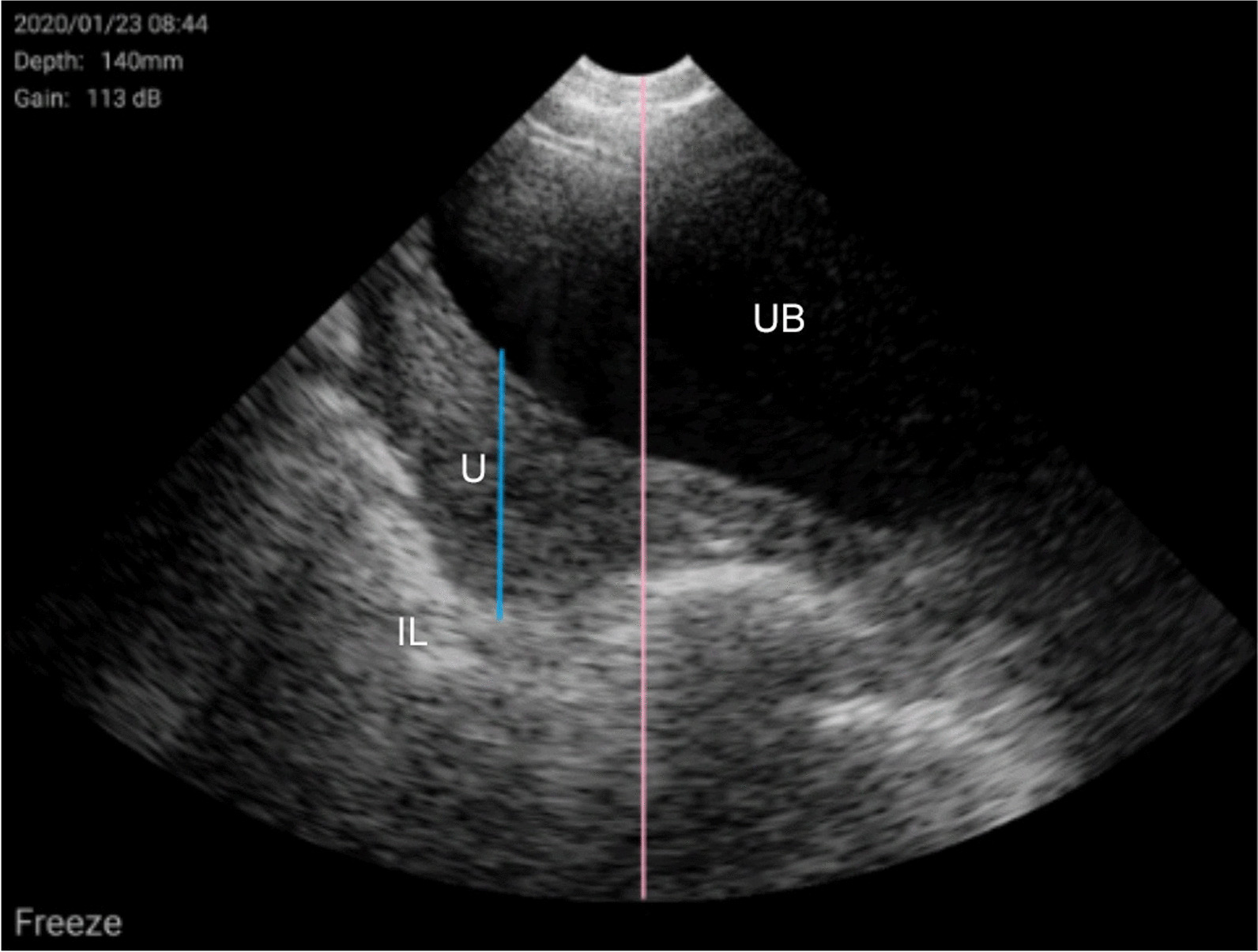
Prepubertal gilt (V2/W3). Urine bladder (UB), intestinal loop (IL) and uterus (U).UB (completely anechoic structure) shows a great volume of urine. The gas hyperechogenicity allows distinguishing IL (white line). U appears as a homogeneous and echogenic structure situated between UB and IL. The U height (in blue) is smaller than two-thirds of the total height of the image (in pink); this means that U is not yet fully developed because uterine inactivity, characteristic of a prepubertal gilt

Figures 6 and 7 show ultrasonography images from V2/W3.

Results from ultrasonography tests are shown in Table 4. One gilt could not be examined by the V2 technician using the W3 equipment.

**Table 4:** Characteristics of ultrasonography tests

Technician/Equipment		Reference standard		Total
		PRE	PUB	
V1/Delta	PRE	23	2	25
	PUB	1	44	45
	Total	24	46	70
V1/W3	PRE	23	5	28
	PUB	1	41	42
	Total	24	46	70
V2/W3	PRE	19	9	28
	PUB	4	37	41
	Total	23	46	69

Cohen´s κ for V1 using the two types of equipment (Delta and W3) was 0.848 (*P* < 0.001), indicating very good concordance. Good concordance (0.668) was found for V1 and V2 using the W3 equipment (Cohen´s κ = 0.668; *P* < 0.001).

The time required for V1/W3 (12.57 ± 11.143 min) was significantly shorter than for both V1/Delta (17.74 ± 10.754 min; *P* = 0.002) and V2/W3 (20.20 ± 15.694 min; *P* = 0.004). No significant differences were found between V1/ Delta and V2/W3 (*P* = 0.833).

Logistic regression models, successively adjusted, are shown in Table 5. All of them were statistically significant (*P* < 0.001), demonstrating a good model fit. For model I, vaginal length, body condition, backfat and live weight (estimated) were used as independent variables. Model II added P4 as independent variable. Models III, IV and V added results from ultrasonography (one technician/ equipment in turn) as independent variables. Finally, models VI and VII included only results from V1/W3 and V2/W3, respectively. Table 5 shows which independent variables were chosen as better predictors in each model. As can be seen, in models II and V two independent variables with *P* value > 0.05 were kept in the best model fit (backfat and P4, respectively); in both models, inclusion of these variables improved fit and explained the percentage of prediction variation.

**Table 5:** Models of multiple logistic regressions

Model	Variable	Coefficient (β)	Standard error	Wald χ^2^	*P* Value	Odds ratio	95% CI		Variables not in the equation
							Lower	Upper	
I	Intercept	−8.666	2.365						Body condition, live weight (estimated)
	Vaginal length (cm)	0.22	0.083	7.096	0.008	1.247	1.060	1.466	
	Backfat (mm)	0.511	0.155	10.832	0.001	1.666	1.229	2.258	
II	Intercept	−5.036	1.525						Body condition, live weight (estimated), vaginal length
	Backfat (mm)	0.338	0.173	3.816	0.051	1.402	0.999	1.968	
	P4 (ng/ml)	1.907	0.955	3.984	0.046	6.731	1.035	43.768	
III	Intercept	3.761	1.012						Body condition, live weight (estimated) vaginal length, backfat, P4
	V1 /DELTA	−6.204	1.252	24.563	< 0.001	0.002	0.000	0.024	
IV	Intercept	−7.645	4.243						Body condition, live weight (estimated) backfat, P4
	Vaginal length (cm)	0.544	0.234	5.420	0.020	1.723	1.090	2.725	
	V1/W3	−7.150	2.224	10.334	0.001	0.001	0.000	0.061	
V	Intercept	−0.341	1.396						Body condition, live weight (estimated), vaginal length, backfat
	P4 (ng/ml)	1.279	1.115	1.314	0.252	3.592	0.403	31.969	
	V2/W3	−2.993	0.991	9.127	0.003	0.050	0.007	0.349	
VI	Intercept	3.714	1.012						
	V1/W3	−5.240	1.126	21.653	< 0.001	0.005	0.001	0.048	
VII	Intercept	2.225	0.526						
	V2 /W3	−2.972	0.664	20.037	< 0.001	0.051	0.014	0.188	

Initially, model I considered morphological characteristics easily measurable in farm (external characteristics diagnosis) as independent variables: vaginal length and backfat were chosen as best pubertal predictors. When progesterone concentration was considered together with external characteristics, only backfat and P4 were chosen (model II). Once results from V1/Delta were considered, only this variable was chosen (model III). However, when results from V1/W3 and V2/W3 were considered, best fit models also includes vaginal length and P4, respectively (models IV and V). Models including only results from V1/W3 and V2/W3 also were significant (models VI and VII).

Table 6 shows the accuracy parameters of the seven logistic regression models (Nagelkerke’s R^2^, Sensitivity, Specificity, Positive predictive value, Negative predictive value, Area under the ROC curve).

**Table 6:** Accuracy parameters of the logistic regression models

Model	Parameter					
	Nagelkerke’s R^2^	Sensitivity (95%IC)	Specificity (95%IC)	Positive predictive value (95%IC)	Negative predictive value (95%IC)	Area under the ROC curve (95%IC)
I	0.520	0.844 (0.738; 0.950)	0.792 (0.629; 0.954)	0.883 (0.787; 0.979)	0.731 (0.561; 0.901)	0.882 (0.806; 0.959)
II	0.722	0.867 (0.767; 0.966)	0.875 (0.743; 1.000)	0.928 (0.850; 1.000)	0.778 (0.621; 0.935)	0.943 (0.890; 0.996)
III	0.846	0.956 (0.897; 1.000)	0.958 (0.878; 1.000)	0.978 (0.935; 1.000)	0.920 (0.814; 1.000)	0.957 (0.900; 1.000)
IV	0.834	0.933 (0.860; 1.000)	0.958 (0.878; 1.000)	0.977 (0.932; 1.000)	0.885 (0.764; 1.000)	0.972 (0.937; 1.000)
V	0.780	0.955 (0.895; 1.000)	0.826 (0.671; 0.981)	0.915 (0.835; 0.995)	0.905 (0.780; 1.000)	0.970 (0.933; 1.000)
VI	0.746	0.891 (0.801; 0.981)	0.958 (0.878; 1.000)	0.976 (0.929; 1.000)	0.821 (0.680; 0.962)	0.925 (0.854; 0.996)
VII	0.442	0.804 (0.690; 0.919)	0.826 (0.671; 0.981)	0.902 (0.812; 0.992)	0.678 (0.506; 0.850)	0.815 (0.703; 0.897)

Models I and VII, respectively based on external characteristics and V2/W3, showed lowest accuracy values. External characteristics based diagnosis improved when progesterone concentration was considered with backfat (model II). Also, V2/W3 improved when considered with P4 for pubertal diagnoses (model V). Results from V1/W3 became more accurate when vaginal length was also considered (model IV). Finally, best accuracy was obtained only from V1/Delta (model III); even though area under ROC curve was lower for model III than for models IV and V, their 95% IC widely overlapped.

## Discussion

The onset of puberty is a complex physiological process where endocrine and physical factors are associated to achieve sexual maturation. The age at puberty onset is in part controlled by individual genetics (moderately heritable, r = 0.38) and can show individual variability [39, 40].

Due to the failure of puberty diagnosis, about 30–60% of gilts are culled, causing a severe economic impact in modern commercial farms [41, 42]. This percentage of culled gilts could be reduced by an improved techniques and effectiveness of estrus detection.

In the present study, postmortem examination was used as a reference standard to represents the actual situation or as close to it as current measures allow. In PRE gilts, the ovaries are characterized as honeycomb (1-3 mm follicles), grape (up to 6 mm) or intermediate type [13, 43]. In the PRE gilts, follicles seem to be recruited in waves, but only grow to 6 mm in size before undergoing atresia [12]. Therefore, distribution of follicle size was similar in both groups up to 6 mm; follicles larger that 6 mm were only present in PUB gilts. The number of *corpus luteum* and *corpus albicans* in both ovaries in PUB gilts are compatible with a normal first cycle estrus [13].

PRE and PUB gilts clearly differed in the development of different sections of the genital tract, as previously described for length of uterine [44, 45] and uterine sections [25, 46]. Furthermore, increased follicular development was accompanied by increased size of all uterus sections [25, 46].

Both internal (breed, body weight, backfat) and management (nutrition, boar contact, surroundings) factors control puberty in gilts, mediated by the endocrine-reproductive axis [28]. Gilts with a high growth rate attained puberty earlier than those with low growth rate [47, 48]. Body weight and backfat have an impact on gilt reproduction. Releasing of gonadotropins and maturation of ovarian follicles depend on body weight and fat [49], growth rate and age [50, 51]. The particular effects of these factors are difficult to ascertain, but slow growing gilts are lighter and show both thinner backfat at selection and delayed puberty, being more likely to be culled [52].

Backfat has been related with puberty onset [53]. Gilts with high backfat (17.8 mm), fed ad libitum, reached puberty at 198 days of age, whereas those with low backfat (14.7 mm), restricted to 80% feed, attained puberty at 203 days of age [54]. Heritability for age at puberty (h^2^ = 0.3) has been reported as slightly higher than for other reproductive traits [54]; therefore, the selection of replacement gilts on the basis of backfat could contribute to excellent reproductive performance of the herd. Tummaruk et al. [48] showed that gilts had their first estrus at 195 days of age with 106 kg body weight and 11 mm backfat, on average, but marked differences in the weight and backfat were found.

Under farm condition, objective assessment of body condition is not easy. Assessment of body condition is based on visual examination of fatness, scores ranging from 1 to 5. Given that this evaluation relies on personal scoring skills, it is regarded as an imprecise and subjective method [9]. Also, live weight was not directly measured, but estimated from carcass weight, as mentioned in Material and methods. The exclusion of these variables from the predictive equation (model I) could be explained by their low accuracy.

On the other hand, the relationship between age, body weight, body composition, and puberty onset is controversial. Dietary treatments do not seem to affect pubertal age [55]. As reviewed by Rauw et al. [56], gilts with a greater lean percentage had a delayed onset of puberty, and negative genetic correlations have been reported between growth rate and estrus signs at puberty [54, 57]. Dietary conditions and exposure to mature boards was more related with puberty onset in gilts than minimum threshold amount of body tissues or a specific rate of body reserves [58]. These facts would explain for the lower accuracy of model I, at least in the described farm conditions.

Eliasson [59] highlighted the importance of progesterone analysis in puberty diagnosis. Progesterone concentration only increases after puberty, following the formation of the first *corpus luteum* [27, 28]. Therefore, gilts showing progesterone concentration > 2ngmL-1 will be considered as pubertal gilts but below this value they will be classified as prepubertal ones [29, 60]. We found than P4 greatly differed between pubertal and prepubertal gilts, with higher values for pubertal ones; P4 is a good puberty marker. As stated before (see “Statistical analysis”), P4 was proposed as independent variable only for models II- V. The stepwise procedure used for fitting logistic regression models chose independent variables for remaining in the model on the basis of their statistical significance, taking into account the effects of the other independent variables included in the model. Hence, P4 was retained only in models II and V, because including this variable improved models fit. Therefore, better accuracy parameters were obtained when P4 was added to models where less powerful independent variables were previously included (backfat and V2/W3, respectively). However, when V1/Delta and V1/W3 were retained in models III and IV respectively, power for detecting puberty was so high than no benefit was accomplished by including P4 in these models.

Ultrasonography has been recommended as a reliable and less laborious method for puberty in gilts for both research and farm, reporting an accuracy of 95–100% (percent of gilts correctly classified by ultrasonography as PRE or PUB) when both uterus and ovaries are examined [29, 61]. Ultrasonography can detect ovaries and their structures; even small follicles can be due to its anechoic appearance [62]. In the transition from PRE to PUB status, the uterus grows strongly and the uterine horn diameters increase [61]. Therefore, while the prepubertal uterus is a small structure needing more time for visualization, the pubertal uterus can be seen quickly [29]; even subjective determination of pubertal status in gilts, as described above, would be reliable for puberty diagnosis.

The present work compares different procedures based on their ability to detect puberty in gilts on farm conditions, assessed by the accuracy parameters (Nagelkerke’s R^2^, Sensitivity, Specificity, Positive predictive value, Negative predictive value, Area under the ROC curve). These parameters can be used as guidance for choosing the diagnosis procedure and this is an important result of this work. As shown, the most accurate results were obtained by V1/Delta (model III), followed by V1/W3 when used in conjunction with vaginal length (model IV) and V2 /W3 with progesterone concentration (model V). However, in farm practice, additional factors must be considered in the choice of puberty diagnosis procedure.

We considered two ultrasound equipments with different characteristics. Designed for pregnancy diagnosis at every gestational age, they do not require a specific installation and adapt to any situation. Ultrasound equipment W3® is a commercial device with an affordable cost for all companies in the sector, widely distributed worldwide because it is totally portable and easy to use. The high-resolution ultrasound Mylab Delta® is professional equipment, robust and portable, that needs a qualified technician and has a high cost. Time required for puberty diagnoses depends on both equipment and technician experience, mean values being lower for W3 and experienced technicians.

Implementing ultrasonography diagnosis can be a challenge on some farms due to the necessary investment in equipment purchase and/or basic technician training. Even in best ecographical images, some ambiguity remain for the untrained eye; this is one of the more frequent criticism about these techniques for be used in farms. Therefore, puberty diagnosis based on backfat and progesterone concentration (model II) would be sufficient enough in view of its accuracy values, avoiding new investments.

## Conclusions

Ultrasonography provided better results than other diagnostic techniques, although V2 obtained the worst results. These results highlight the need for experienced technicians. The most accurate results were obtained by V1/Delta: the effectiveness of the ultrasonography changes with technological improvements and with increased expertise of technicians. Results from W3 procedure could be improved when used in conjunction with vaginal length (V1) or progesterone concentration (V2).

Accuracy parameters can be used as guidance for choosing the diagnosis procedure but procedures can also be compared based on ease of administration, cost of administration, and effect on patients (invasiveness, discomfort, convenience). In this sense, ultrasonography equipment is usually present in farms due its use in pregnancy diagnosis and in these cases, ultrasonography is cheaper than progesterone concentration analysis. Also, it causes less discomfort than vaginal length measurement and takes less time when used by an experienced technician. However, ultrasonography diagnosis needs investment in equipment purchase and/or basic technician training. As shown, puberty diagnosis based on backfat and progesterone concentration could be a good alternative, in view of its accuracy values.

## Data Availability

The datasets used and/or analyzed during the current study are available from the corresponding author on reasonable request.

## Abbreviations

P4: Progesterone concentration
V1: Expert technician
V2: Junior technician
Delta: Ultrasound Mylab Delta
W3: Ultrasound W3
PRE: Prepubertal
PUB: Pubertal
SD: Standard deviation
ANOVA: Analysis of variance
ANCOVA: Analysis of covariance
SE: Standard error
CI: Confidents intervals
